# Development and validation of nomograms predicting survival in Chinese patients with triple negative breast cancer

**DOI:** 10.1186/s12885-019-5703-4

**Published:** 2019-06-06

**Authors:** Yaping Yang, Ying Wang, Heran Deng, Cui Tan, Qian Li, Zhanghai He, Wei Wei, Enxiang Zhou, Qiang Liu, Jieqiong Liu

**Affiliations:** 1 Guangdong Provincial Key Laboratory of Malignant Tumor Epigenetics and Gene Regulation, Breast Tumor Center, Sun Yat-sen Memorial Hospital, Sun Yat-sen University, Yanjiang West Road 107#, Guangzhou, 510120 China; 2 Guangdong Provincial Key Laboratory of Malignant Tumor Epigenetics and Gene Regulation, Department of Pathology, Sun Yat-sen Memorial Hospital, Sun Yat-sen University, Guangzhou, China; 3 Department of Breast and Thyroid Surgery, Peking University Shenzhen Hospital, Shenzhen, China; 4 Department of Breast and Thyroid Surgery, the Second Xiangya Hospital, Central South University, Changsha, Hunan China

**Keywords:** Nomograms, Disease-free survival, Overall survival, Triple negative breast cancer, Stromal tumor-infiltrating lymphocytes

## Abstract

**Background:**

Triple negative breast cancer (TNBC) is an aggressive and heterogeneous disease. Nomograms predicting outcomes of TNBC are needed for risk management.

**Methods:**

Nomograms were based on an analysis of 296 non-metastatic TNBC patients treated at Sun Yat-sen Memorial Hospital from 2002 to 2014. The end points were disease-free survival (DFS) and overall survival (OS). Predictive accuracy and discriminative ability were evaluated by concordance index (C-index), area under the curve (AUC) and calibration curve, and compared with the American Joint Committee on Cancer (AJCC) staging system, PREDICT and CancerMath. Models were subjected to bootstrap internal validation and external validation using independent cohorts of 191 patients from the second Xiangya Hospital and Peking University Shenzhen Hospital between 2007 and 2012.

**Results:**

On multivariable analysis of training cohort, independent prognostic factors were stromal tumor-infiltrating lymphocytes (TILs), tumor size, node status, and Ki67 index, which were then selected into the nomograms. The calibration curves for probability of DFS and OS showed optimal agreement between nomogram prediction and actual observation. The C-index of nomograms was significantly higher than that of the seventh and eighth AJCC staging system for predicting DFS (training: 0.743 vs 0.666 (*P* = 0.003) and 0.664 (*P* = 0.024); validation: 0.784 vs 0.632 (*P* = 0.02) and 0.607 (*P* = 0.002)) and OS (training: 0.791 vs 0.683 (*P* = 0.004) and 0.677 (*P* < 0.001); validation: 0.783 vs 0.656 (*P* = 0.006) and 0.606 (*P* = 0.001)). Our nomograms had larger AUCs compared with PREDICT and CancerMath. In addition, the nomograms showed good performance in stratifying different risk groups of patients both in the training and validation cohorts.

**Conclusion:**

We have developed novel and practical nomograms that can provide individual prediction of DFS and OS for TNBC based on stromal TILs, tumor size, node status, and Ki67 index. Our nomograms may help clinicians in risk consulting and selection of long term survivors.

**Electronic supplementary material:**

The online version of this article (10.1186/s12885-019-5703-4) contains supplementary material, which is available to authorized users.

## Background

Breast cancer is the most frequently diagnosed cancer and the leading cause of cancer death in women worldwide. It is a heterogeneous disease, and different subtypes of breast cancer show distinct clinicopathologic features, aggressiveness, response to therapies, as well as survival outcomes. Triple negative breast cancer (TNBC), the most aggressive subtype of breast cancer characterized by lack of expression of estrogen receptor (ER), progesterone receptor (PR) and human epidermal growth factor receptor 2 (HER2), has significantly poorer outcomes than non-TNBC subtypes due to the natural history of this life-threating disease and lack of endocrine and target therapies [1, 2]. Indeed, TNBC is also a heterogeneous disease including several distinct molecular subtypes that differ in biological features, treatment response and prognosis [3]. Nomogram is an useful and convenient tool for cancer patients to quantify and predict risk and prognosis. For breast cancer patients, a lot of prognostication nomograms have been developed and validated based on traditional clinicopathological features [4–14]. However, the prognostic values of these models were only tested in a few cohorts of TNBC subgroup [6, 13, 15]. Furthermore, the majority of these models were developed based on white patients not Asian women, and only one nomogram [13] can predict recurrence risk (most models focused on overall survival or breast cancer-specific survival). Thus, nomograms for predicting recurrence risk and survival outcomes in TNBC are scarce. Stromal tumor-infiltrating lymphocytes (TILs) are recently reported to show important prognostic value in TNBC, both in the adjuvant and neoadjuvant settings [16–19]. Yet, TILs have not been included in any breast cancer prognostic models so far.

In the current study, we aimed to develop nomograms to predict the disease-free and overall survival for non-metastatic TNBC patients using clinicopathological and molecular variables as well as stromal TILs from 296 TNBC patients treated at Sun Yat-sen Memorial Hospital in China. Moreover, we externally validated the prognostic models using independent cohorts of 191 Chinese women from the second Xiangya Hospital and Peking University Shenzhen Hospital.

## Methods

### Patient population and data processing

The training set was based on data from 296 patients with invasive TNBC who meet the inclusion criteria diagnosed and treated at Sun Yat-sen Memorial Hospital from 2002 to 2014. inclusion criteria defined eligible women who were age 18 years or older and had diagnosed non-metastatic invasive breast cancer, had confirmed histology as defined by the American Joint Committee on Cancer (AJCC) (thresholds for defining ER/PR negative were set at less than 1% using immunohistochemical staining), had complete follow-up, availability of tumor samples, no history of previous malignancies (except for primary skin basal cell carcinoma and squamous cell carcinoma). Totally, there were 435 non-metastatic invasive TNBC patients who were age 18 years or older treated at Sun Yat-sen Memorial Hospital from 2002 to 2014. We excluded 52 (12.0%) patients who had incomplete follow-up information, and then removed 84 patients whose tumor samples were not available, as well as 3 women with a history of previous malignancies. For patients who underwent neoadjuvant chemotherapy and had clinically negative axillary lymph nodes, sentinel lymph node biopsy was performed before neoadjuvant therapy. And all of the pathologically node-positive patients received axillary lymph node dissection. An external validation cohort of 191 TNBC women who met the same inclusion criteria was enrolled from the second Xiangya Hospital (*n* = 144) and Peking University Shenzhen Hospital (*n* = 47) between 2007 and 2012. All patients were required to have sufficient information to score all variables in the developed nomograms. Ethical approval was obtained from participating institutions through their respective institutional review boards Ethical approval was obtained from participating institutions through their respective institutional review boards (IRB) (Sun Yat-sen Memorial Hospital Ethics Committee and IRB, Ethics Committee and IRB of Peking University Shenzhen Hospital, and Ethics Committee and IRB of the Second Xiangya Hospital, Central South University), and written informed consent was obtained from study participants.

We retrieve all relevant information on demographic data (age, marital status, family history of breast cancer), clinicopathological features (menstrual status, histological type, grade, tumor size, node status, Ki67 index), and treatment information (surgery type, receiving of radiotherapy, chemotherapy type, chemotherapy regimen) for all of the included patients. In the dataset, some variables (grade, and Ki67 index) contained missing data, which may result in biases. To compensate for this, multiple imputation methods by chained equations [20–22] to account for the missing values of variables was performed before nomogram development and validation. We have created ten multiple imputed-datasets, and variables included in the imputation model were age, marital status, family history of breast cancer, menopausal status, tumor size, node status, stage and sTIL group. The raw stromal TILs and Ki67 values were estimated using a CLIA certified lab. Stromal TILs were evaluated in hematoxylin and eosin (H&E) sections originally sampled from each TNBC included in this study, following the criteria proposed by the International TIL WG [23]. Concisely, all mononuclear cells in the stromal compartment within the borders of the invasive tumor were evaluated and assessed as a percentage value. The scoring report did not include TILs which were outside of the tumor borderline, or around DCIS and normal tissue, or in the necrosis areas. One experienced pathologist has evaluated stromal TILs in all the cases. Ninety-eight randomly selected cases, corresponding to approximate 20% of the study population, were separately annotated by a second pathologist for assessing the inter observer consistency of the readings. The end points were disease-free survival (DFS defined as time from date of diagnosis to the local, regional recurrence, distant metastasis, contralateral breast cancer, death (including non-cancer death) or last contact (June 30th, 2017)) and overall survival (OS, calculated from date of diagnosis to the date of death or last contact (June 30th, 2017)).

### Statistical analysis

Survival curves for distinct variables were generated using the Kaplan-Meier estimates and were compared using log-rank test. Prognostic factors that assessed by univariable Cox analysis were subjected to backward stepwise (which used the Akaike information criterion) Cox proportional regression analysis to identify statistically significant variables (*P* < 0.05) to be included in the final nomograms. Interaction between variables was assessed by adding interaction variable to the Cox model. We tested the interactions between ki67 and node status, ki67 and tumor size, as well as tumor size and node status. For the calibration (modified Hosmer- Lemeshow statistic for survival analysis), the nomograms were then subjected bootstrap method [24] of leave one out prediction 1000 times for internal validation of training cohort and external validation of validation cohort by R statistical software, rms package. The bootstrap resampling for internal validation was performed to reduce the over-fitting bias of the model and obtain the evaluation value of more reliable prediction accuracy of the model. External validation with independent cohorts of 191 women from the second Xiangya Hospital and Peking University Shenzhen Hospital was also performed. The predictive accuracy and discriminative ability of nomograms were determined by concordance index (C-index) (C index is actually a generalization of the area under the ROC curve [25]), area under the curve (AUC) and calibration curves. We compared the predictive accuracy and discriminative ability of our nomograms with the seventh and eighth AJCC staging system, and the classical PREDICT [6] and CancerMath models [9]. Comparison between two different models was according to previously described methods [26]. We compared the predicted survival with observed actual survival to calibrate the nomograms for 3-, and 5-year DFS and OS. Furthermore, we determined the cutoff values of the predicted scores for differentiating patients to low-risk, intermediate-risk, and high-risk groups using the X-tile software program (Yale University, New Haven, CT, USA [27]) based on the maximal chi-square test by grouping all the patients into distinct risk groups after sorting by total score. To avoid the problem of multiple cut-point selection, X-tile can produce corrected *P* values using several Monte Carlo simulations. And the respective Kaplan-Meier curves were then delineated. Statistical analyses and modeling were performed using STATA (version 13; Stata Co., College Station, TX), and R software packages. All statistical tests were two-sided, and statistical significance was defined as *P* < 0.05.

## Results

### Study population characteristics

The training population included 296 invasive non-metastastic TNBC women treated at Sun Yat-sen Memorial Hospital with a median follow-up of 52.5 months. There were 78 DFS events, and 46 deaths during the follow-up period for training cohort. Independent validation cohorts were compromised of 191 women diagnosed with operable invasive TNBC in the second Xiangya Hospital (*n* = 144) and Peking University Shenzhen Hospital (*n* = 47) over a median follow-up of 68 months. A total of 51 DFS events and 32 deaths occurred in the validation population. Some collected variables (grade, and Ki67 index) contained missing data (less than 20%), so multiple imputation was performed before nomogram development and validation to account for the missing values of these variables. Demographic and clinicopathological characteristics of patients in the training and validation cohorts before and after multiple imputations are shown in Table 1.

**Table 1:** Demographic and clinicopathological characteristics of patients in the training and validation cohorts before and after multiple imputation

Characteristics	Trainingcohort before imputation		Training cohort after imputation		Validation cohort before imputation		Validation cohort after imputation		*P*
	*N*	%	*N*	%	*N*	%	*N*	%	
Age									0.302
Median (range)	48 (26–88)		48 (26–88)		46 (20–87)		46 (20–87)		
≤35	35	11.8	35	11.8	25	13.1	25	13.1	
36–50	138	46.6	138	46.6	100	52.4	100	52.4	
>50	123	41.6	123	41.6	66	34.6	66	34.6	
Marital status									
Married	281	94.9	281	94.9					
Unmarried	15	5.1	15	5.1					
Family history of breast cancer									
Yes	34	11.5	34	11.5					
No	262	88.5	262	88.5					
Menopausal status									
Premenopausal	174	58.8	174	58.8					
Postmenopausal	122	41.2	122	41.2					
Histology									0.329
Ductal	274	92.6	274	92.6	172	90.1	172	90.1	
Lobular/other	22	7.4	22	7.4	19	9.9	19	9.9	
Grade									0.890
I	11	3.7	15	5.1	11	5.8	11	5.8	
II	49	16.6	54	18.2	37	19.4	37	19.4	
III	181	61.1	227	75.0	143	74.9	143	74.9	
Unknown	55	18.6	0	0.0					
Tumor size									0.414
≤2 cm	152	51.4	152	51.4	98	51.3	98	51.3	
2-5 cm	120	40.5	120	40.5	71	37.2	71	37.2	
>5 cm	24	8.1	24	8.1	22	11.5	22	11.5	
Node status									0.630
N0	172	58.1	172	58.1	108	56.5	108	56.5	
N1	73	24.7	73	24.7	54	28.3	54	28.3	
N2 + N3	51	17.2	51	17.2	29	15.2	29	15.2	
Stage									0.958
I	97	32.8	97	32.8	65	34.0	65	34.0	
II	141	47.6	141	47.6	89	46.6	89	46.6	
III	58	19.6	58	19.6	37	19.4	37	19.4	
Ki67 index									0.150
<40%	111	37.5	126	42.6	86	45.0	94	49.2	
≥40%	139	47.0	170	57.4	92	48.2	97	50.8	
Unknown	46	15.5	0	0.0	13	6.8	0	0.0	
sTIL group (%)									0.146
0–9	61	20.6	61	20.6	25	13.1	25	13.1	
10–19	79	26.7	79	26.7	62	32.5	62	32.5	
20–49	85	28.7	85	28.7	60	31.4	60	31.4	
≥50	71	24.0	71	24.0	44	23.0	44	23.0	
Surgery type									
Mastectomy	135	45.6	135	45.6					
Lumpectomy	161	54.4	161	54.4					
Radiotherapy									
Yes	197	66.6	197	66.6					
No	99	33.4	99	33.4					
Chemotherapy type									
Neoadjuvant	62^a^	20.9	62^a^	20.9	34^b^	17.8	34^b^	17.8	0.515
Adjuvant	222	75.0	222	75.0	146	76.4	146	76.4	
No chemotherapy	12	4.1	12	4.1	11	5.8	11	5.8	
Chemotherapy regimen									
anthracycline-based	70	24.6	70	24.6	44	24.4	44	24.4	0.622
taxane-based	72	25.4	72	25.4	39	21.7	39	21.7	
anthracycline& taxane -based	142	50.0	142	50.0	97	53.9	97	53.9	

Abbreviations: sTIL, stromal tumor-infiltrating lymphocyte; ^a^ The group contained 10 patients who received both neoadjuvant and adjuvant chemotherapy. ^b^ The group contained 5 patients who received both neoadjuvant and adjuvant chemotherapy

### Prognostic nomogram for DFS

**Fig. 1:**
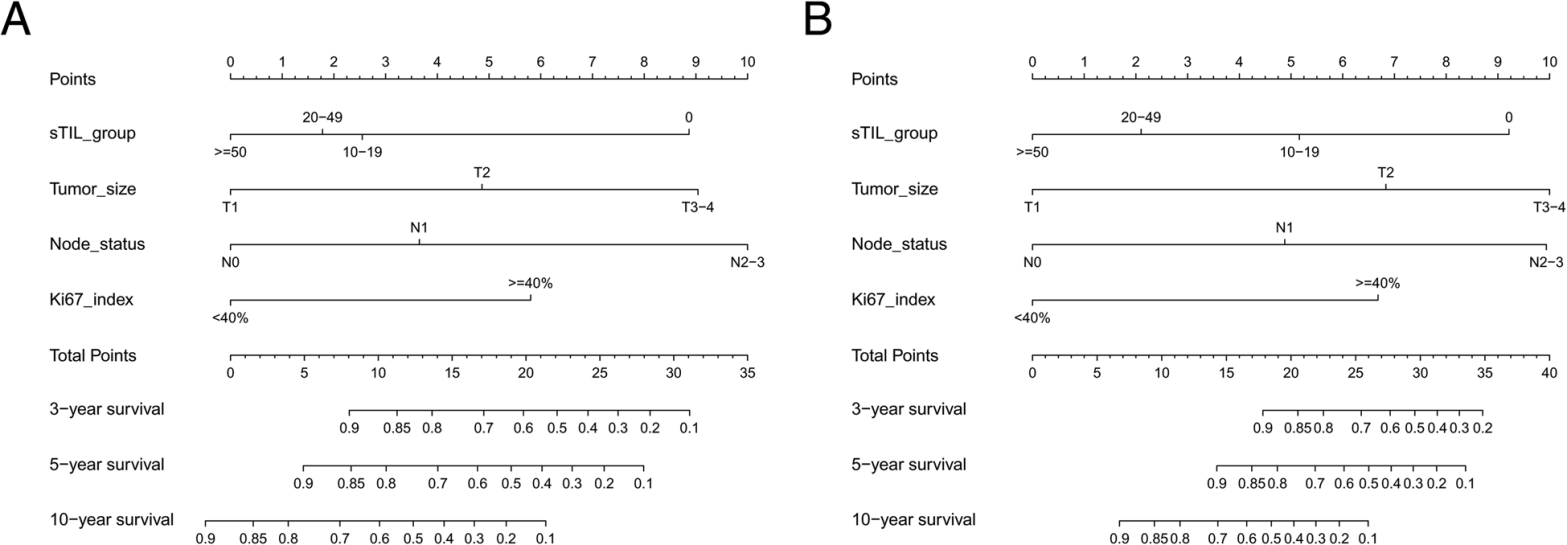
Prognostic nomograms for predicting (**a**) DFS and (**b**) OS of patients with non-metastatic TNBC (When using these nomograms, individual patient’s value will be located on each variable axis, and a line will be drawn to determine the scores received for each variable value. Sum of the scores will then be located on the Total Points axis. According to the scores, we may predict the 3 year-, 5 year-, and 10 year-DFS or OS for this individual)

**Fig. 2:**
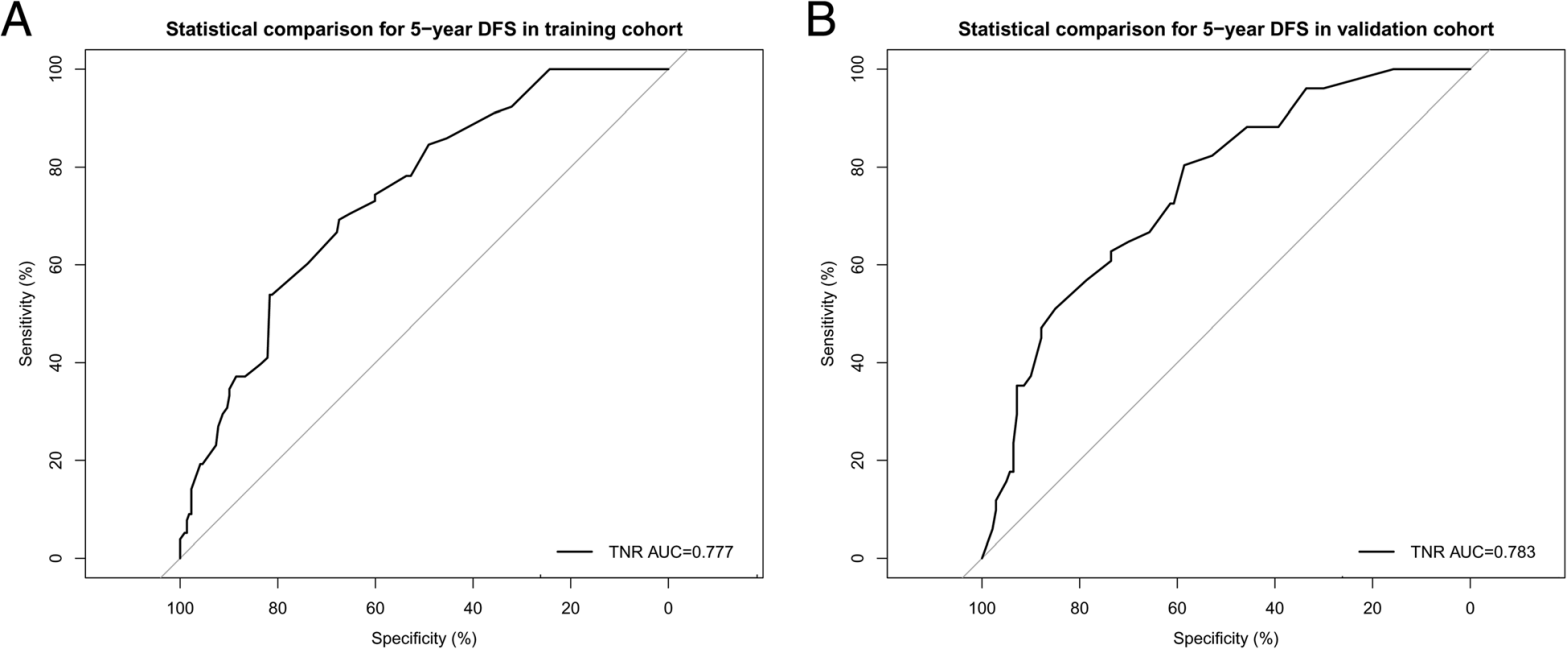
Discriminatory accuracy for predicting DFS assessed by receiver operator characteristics analysis calculating AUC. 5-year DFS in the **a**) training cohort and **b**) validation cohort. TNR = triple-negative recurrence; AUC = area under the curve

In the training set, DFS curves for different demographic, clinicopathological and treatment factor values were generated by the Kaplan-Meier estimates and were compared by log-rank test. The variables that selected in the final multivariable Cox regression model were stromal TILs, tumor size, node status, and Ki67 index (Table 2). A nomogram that incorporated these four prognostic variables was then developed (Fig. 1a), and we named this nomogram as triple-negative recurrence (TNR). Each subtype within these variables was assigned a score on the point scale (Additional file 1: Table S1.). Briefly, we can put the specific values of a TNBC patient into the TNR nomogram, and then calculated a score for this patient. According to the score, we may predict the 3 year- and 5 year-DFS for this individual. Ideal concordance in AUC was observed for the nomogram in both training and validation cohort with C-index of 0.743 and 0.784, respectively, and AUC of 0.777and 0.783 (Fig. 2), respectively.

**Table 2:** Univariable and multivariable analysis of training set for DFS

	Univariable an- alysis *P*	Multivariable analysis			Selected factors for building the nomogram		
Variable		HR	95% CI	*P*	HR	95% CI	*P*
Grade	0.024						
I		Ref					
II		0.889	0.108-7.308	0.913			
III		1.477	0.193-11.297	0.707			
Tumor size	<0.001						
≤2cm		Ref			Ref		
2-5cm		2.010	1.168-3.456	0.012	1.912	1.156-3.163	0.012
>5cm		3.481	1.578-7.675	0.002	3.319	1.526-7.217	0.002
Node status	<0.001						
N0		Ref			Ref		
N1		1.464	0.789-2.716	0.227	1.628	0.933-2.843	0.086
N2+N3		3.613	2.043-6.390	<0.001	3.784	2.173-6.589	<0.001
Ki67 index	0.018						
<40%		Ref			Ref		
≥40%		1.977	1.179-3.314	0.010	2.166	1.310-3.583	0.003
sTIL group (%)	0.002						
0-9		Ref			Ref		
10-19		0.436	0.238-0.801	0.007	0.432	0.240-0.778	0.005
20-49		0.383	0.200-0.734	0.004	0.390	0.207-0.734	0.004
≥50		0.305	0.148-0.631	0.001	0.308	0.154-0.616	0.001
Surgery type	0.247						
Mastectomy							
Lumpectomy							
Radiotherapy	0.144						
Yes							
No							
Age	0.886						
≤35							
36-50							
>50							
Marital status	0.753						
Married							
Unmarried							
Family history of breast cancer	0.785						
Yes							
No							
Menopausal status	0.911						
Premenopausal							
Postmenopausal							
Histology	0.655						
Ductal							
Lobular/other							
Chemotherapy type	0.134						
Neoadjuvant							
Adjuvant							
No chemotherapy							
Chemotherapy regimen	0.0019						
anthracycline-based		Ref					
taxane-based		1.239	0.667-2.300	0.497			
anthracycline& taxane -based		0.791	0.444-1.409	0.426			

Abbreviations: *DFS* disease-free survival, *HR* hazard ratio, *CI* confidence interval, *Ref* reference, *sTIL* stromal tumor-infiltrating lymphocyte

### Prognostic nomogram for OS

**Fig. 3:**
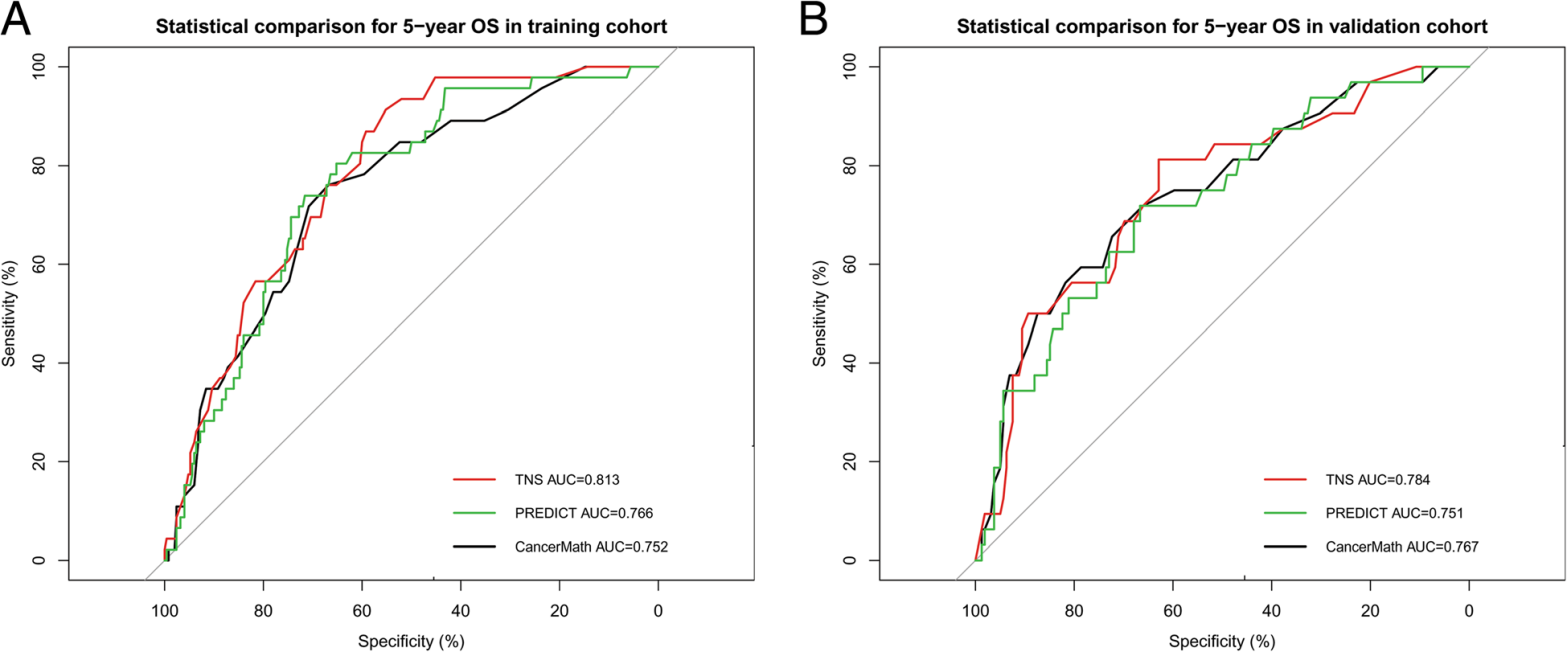
Discriminatory accuracy for predicting OS assessed by receiver operator characteristics analysis calculating AUC. 5-year OS in the **a**) training cohort and **b**) validation cohort. TNS = triple-negative survival; AUC = area under the curve

In the training set, OS curves for different demographic, clinicopathological and treatment variable values (the same variables in the DFS nomogram initiating) were generated. The variables in the final multivariable Cox regression model were stromal TILs, tumor size, node status, and Ki67 index (Table 3). A nomogram that incorporated these four prognostic variables was then developed (Fig. 1b), and the model was named as triple-negative survival (TNS). Each subtype within these variables was assigned a score on the point scale (Additional file 1: Table S1.). Briefly, when using the nomogram, we can put the specific values of a TNBC patient into the TNS nomogram, and then calculated a score for this patient. According to the score, we may predict the 3 year- and 5 year-OS for this individual. Ideal concordance in AUC was observed for the nomogram in both training and validation cohort with C-index of 0.791 and 0.783, respectively, and AUC of 0.813 and 0.784 (Fig. 3), respectively.

**Table 3:** Univariable and multivariable analysis of training set for OS

	Univariable an- alysis *P*	Multivariable analysis			Selected factors for building the nomogram		
Variable		HR	95% CI	*P*	HR	95% CI	*P*
Grade	0.032						
I		Ref					
II		0.151	0.014–1.605	0.117			
III		0.461	0.055–3.876	0.476			
Tumor size	<0.001						
≤2 cm		Ref			Ref		
2-5 cm		3.226	1.541–6.755	0.002	2.981	1.516–5.864	0.002
>5 cm		4.704	1.587–13.939	0.005	4.916	1.705–14.174	0.003
Node status	<0.001						
N0		Ref			Ref		
N1		2.121	0.941–4.778	0.070	2.183	1.050–4.559	0.037
N2 + N3		4.545	2.086–9.902	<0.001	4.900	2.328–10.312	<0.001
Ki67 index	0.018						
<40%		Ref			Ref		
≥40%		2.778	1.339–5.763	0.006	2.906	1.440–5.863	0.003
sTIL group (%)	0.025						
0–9		Ref			Ref		
10–19		0.578	0.269–1.240	0.159	0.523	0.252–1.085	0.082
20–49		0.272	0.108–0.686	0.006	0.320	0.134–0.767	0.011
≥50		0.217	0.075–0.629	0.005	0.230	0.082–0.645	0.005
Surgery type	0.198						
Mastectomy							
Lumpectomy							
Radiotherapy	0.315						
Yes							
No							
Age	0.392						
≤35							
36–50							
>50							
Marital status	0.588						
Married							
Unmarried							
Family history of breast cancer	0.274						
Yes							
No							
Menopausal status	0.107						
Premenopausal							
Postmenopausal							
Histology	0.224						
Ductal							
Lobular/other							
Chemotherapy type	0.374						
Neoadjuvant							
Adjuvant							
No chemotherapy							
Chemotherapy regimen	0.046						
anthracycline-based		Ref					
taxane-based		1.551	0.682–3.528	0.296			
anthracycline& taxane -based		0.781	0.371–1.643	0.514			

Abbreviations: *OS* overall survival, *HR* hazard ratio, *CI* confidence interval, *Ref* reference, *sTIL* stromal tumor-infiltrating lymphocyte

### Calibration of nomograms and comparison with AJCC staging、PREDICT and CancerMath

An acceptable agreement of the calibration plots was found both in the training and validation cohorts between the model prediction and actual data for 3-, and 5-year DFS and OS (Additional file 2: Figure S1). In the training cohort, the C-index for our model to predict DFS (0.743, 95% CI 0.692–0.794) was significantly better than that of the seventh and eighth AJCC TNM staging system (0.666, 95% CI 0.611–0.721, *P* = 0.003; 0.664, 95% CI 0.605–0.723, *P* = 0.024); and the C-index to predict OS (0.791, 95% CI 0.735–0.847) was statistically greater than that of the TNM systems (0.683,95% CI 0.613–0.753, *P* = 0.004; 0.677, 95% CI 0.606–0.748, *P* < 0.001) as well. Similarly, in the validation cohort, the C-index of our model to predict DFS (0.784, 95% CI 0.724–0.844) was much higher than that of the seventh and eighth TNM systems (0.632, 95% CI 0.518–0.746, *P* = 0.02; 0.607, 95% CI 0.554–0.660, *P* = 0.002); and the C-index to predict OS was also better for our nomogram prediction (0.783, 95% CI 0.705–0.861) than for the TNM systems prediction (0.656, 95% CI 0.516–0.796, *P* = 0.006; 0.606, 95% CI 0.535–0.677, *P* = 0.001). Furthermore, we compared the predictive accuracy and discriminative ability of our nomograms with two classical breast cancer models. The AUC for OS was0.813 in the training and 0.784 in the validation cohort, respectively, which was larger than the AUCs of 0.752 and 0.767 in training and 0.766 and 0.751 in validation for PREDICT and CancerMath, respectively (Fig. 3).

### Performance of the nomogram in stratifying risk of patients

**Fig. 4:**
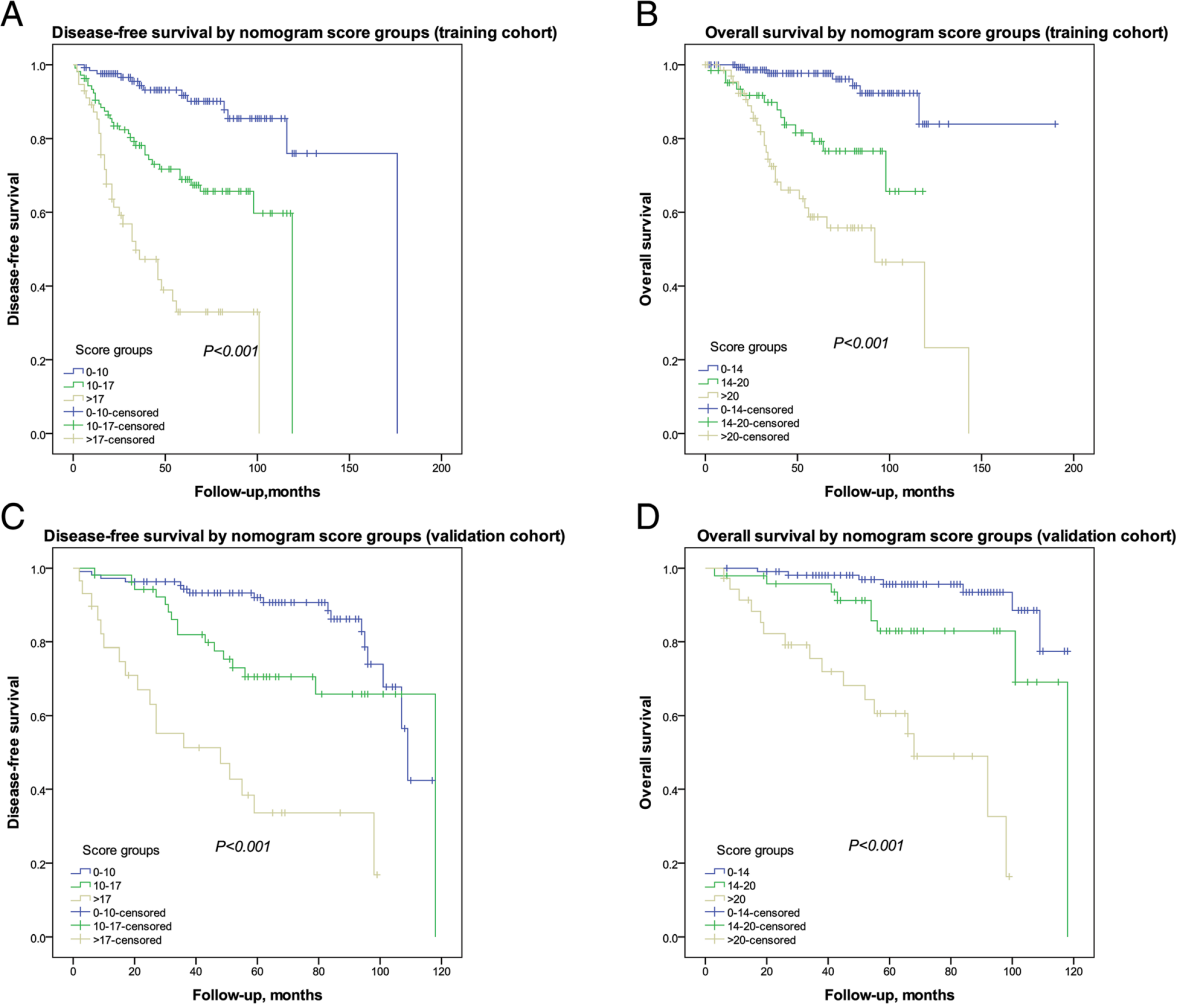
Risk group stratification in the training and validation cohort. DFS curves of patients in the **a**) training cohort and **c**) validation cohort by nomogram (TNR) score groups; OS curves of patients in the **b**) training cohort and **d**) validation cohort by nomogram (TNS) score groups. TNR = triple-negative recurrence; TNS = triple-negative survival

We then defined the cutoff values using X-tile software program by grouping patients in the training cohort into three groups after sorting by total DFS or OS score (Additional file 1: Table S1.). Each group showed significantly different survival outcomes (Additional file 1: Table S1., Fig. 4a and b). These cutoff values also well differentiated patients in the validation cohort to low-risk, intermediate-risk, and high-risk groups with extremely distinct prognosis (Fig. 4c and d).

## Discussion

To the best of our knowledge, this is the first study that incorporates stromal TILs into clinicopathological variables in predicting prognosis for TNBC patients. Our nomograms, named as TNR and TNS, which were developed using the Chinese TNBC patients treated at Sun Yat-sen Memorial Hospital, showed AUCs of 0.777 for DFS and 0.813 for OS in the training cohort. The discriminatory accuracy of TNR/TNS was then validated in independent external validation patient population from the second Xiangya Hospital and Peking University Shenzhen Hospital by AUCs of 0.783 for DFS and 0.784 for OS. In addition, our nomograms showed significantly higher C-index than that of the seventh and eighth AJCC TNM staging system in predicting DFS and OS; and larger AUCs compared with the classical prognostic models including PREDICT and CancerMath, although the improvement was little.

When assessing the outcomes and risk of breast cancer, predictive nomograms are useful tools. Lots of such models have been developed based on clinicopathological and receptors statuses [4–14]. Nevertheless, majority of these models were developed based on white patients from American and European countries, and many nomograms focused on OS or breast cancer specific survival (BCSS), but not DFS. One nomogram [13] can predict recurrence risk, but it may not be generalizable to external populations because it was developed using patients from a famous large single institution (MD Anderson Cancer Center) that may bring potential referral and therapeutic bias. As we known, the clinicopathological features and prognosis of breast cancer may vary by race/ethnicity. For instance, the average age of onset for Asian women was approximately 10 years younger than that for western women [28–33]. Therefore, the majority of these models that were developed based on western patients may have limited value in Asian breast cancer patients. There is a nomogram developed from Taiwanese women, however, it can only predict OS for patients treated with mastectomy [12]. Furthermore, the prognostic values of these existing models were only tested in a few cohorts of TNBC [6, 13, 15], which is a heterogeneous disease comprised of several distinct subtypes with totally different prognosis. A potential predictive model for TNBC based on simple sum of ≥4 positive lymph nodes, positive Cathepsin-D expression and Ki-67 index ≥20% has been reported previously [34]. However, the score for each variable was not well justified, and the model included patients only from a single institution. Moreover, it showed smaller AUCs for predicting survival both in the training (0.696) and validation set (0.717) compared with our model.

Further, compared with existing nomograms, TNR/TNS incorporated several new and potentially universal predictive or prognostic factors for TNBC including stromal TILs and Ki67 index. The prognostic significance of TILs in TNBC has been recently demonstrated in a number of randomized clinical trials, both in the neoadjuvant and adjuvant settings [16–19]. The International TIL Working Group released detailed guidelines in 2014 for harmonizing TILs assessment in routine samples [23]. In this study, we assessed the stromal TILs strictly by applying the recent International TILWG guidelines. For the first time, we developed and validated nomograms predicting outcomes in TNBC by incorporating stromal TILs into the models. Additionally, previous studies have demonstrated that TNBC with higher Ki-67 index is associated with larger tumor size, more positive nodes, and worse prognosis [35, 36]. Our findings suggested that Ki-67 index ≥40% may be adequate to demonstrate an association with recurrence and unfavorable survival in TNBC.

Despite above strengths, our nomograms are limited by the retrospective nature of data collection and relatively small sample size. Some of the calibration plots for the validation cohort were less than ideal, which is another limitation of this study. Also, the TNR and TNS were based on Chinese TNBC patients, therefore it is not clear whether they can be applied to western patient cohorts or not. Further efforts on prospective data collection, larger patient cohorts, and validation in other geographic patient populations are needed to improve our nomograms.

## Conclusions

We have developed and validated novel, well-calibrated nomograms for predicting DFS and OS in non-metastatic Chinese TNBC patients by including stromal TILs for the first time. These prognostic nomograms can help clinicians in risk consulting/management and selection of long term survivors among TNBC patients. Additional studies are required to identify whether they can be applied to other geographic patient populations.

## Additional files

Additional file 1: **Table S1.** The calibration curves for predicting patient survival at each time point in the training and validation cohort. A) 3-year and B) 5-year DFS in the training cohort; C) 3-year and D) 5-year OS in the training cohort; E) 3-year and F) 5-year DFS in the validation cohort; G) 3-year and H) 5-year OS in the validation cohort. (TIF 3422 kb)

Additional file 2: **Figure S1.** Point assignment and prognostic score for DFS and OS. (DOCX 15 kb)

## Abbreviations

AJCC: American Joint Committee on Cancer
AUC: area under the curve
C-index: concordance index
DFS: disease-free survival
ER: estrogen receptor
H&E: hematoxylin and eosin
HER2: human epidermal growth factor receptor 2
OS: overall survival
PR: progesterone receptor
ROC: receiver operating characteristic
TILs: tumor-infiltrating lymphocytes
TNBC: Triple negative breast cancer
TNR: triple-negative recurrence
TNS: triple-negative survival
