# Supplementary figures and images for: Development and validation of nomograms predicting survival in Chinese patients with triple negative breast cancer
Source: BMC Cancer. 2019 Jun 6;19:541. doi: 10.1186/s12885-019-5703-4 (PMC6555047; doi:10.1186/s12885-019-5703-4)

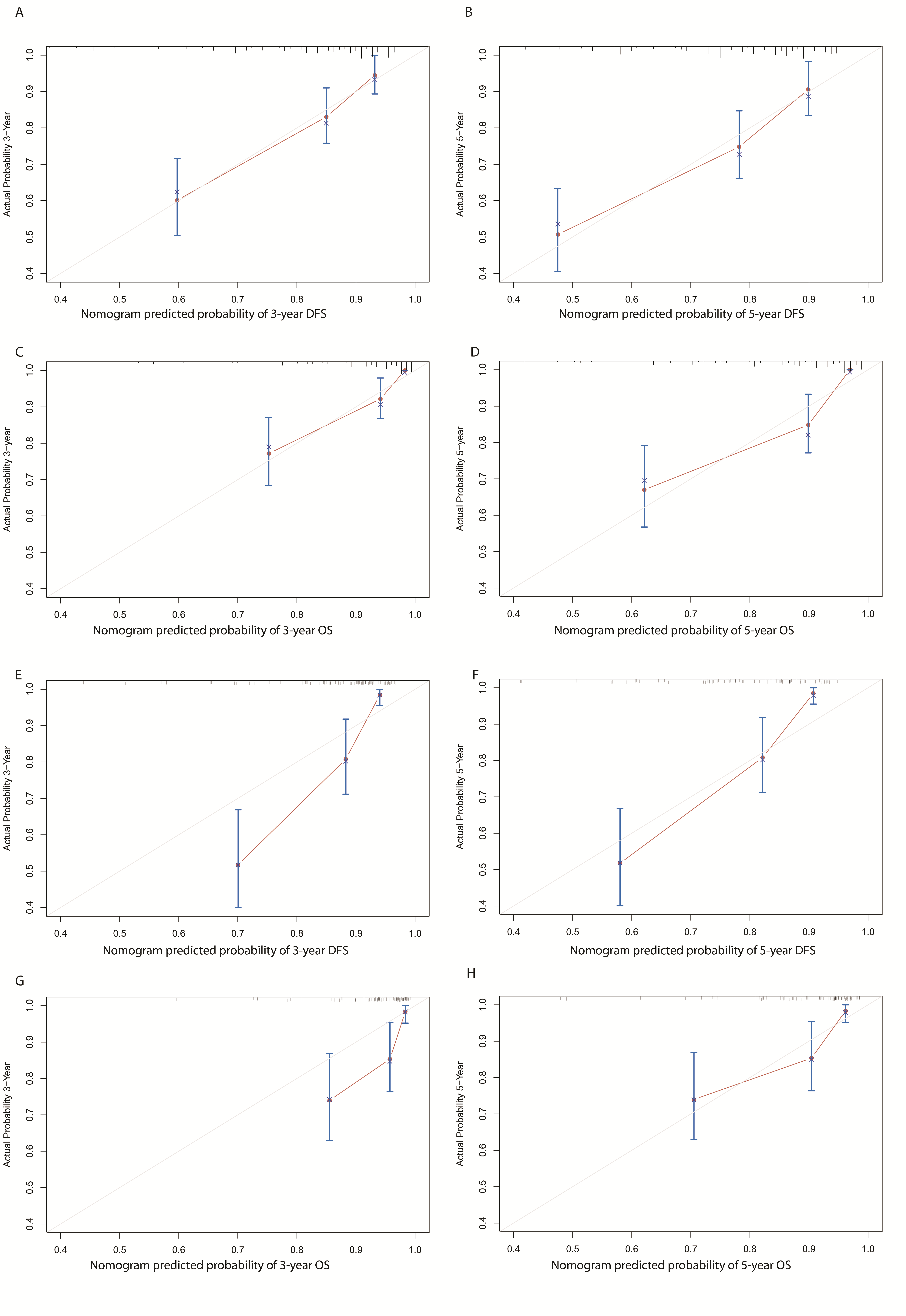

Supplement: Supplementary file 1 — Table S1. The calibration curves for predicting patient survival at each time point in the training and validation cohort. A) 3-year and B) 5-year DFS in the training cohort; C) 3-year and D) 5-year OS in the training cohort; E) 3-year and F) 5-year DFS in the validation cohort; G) 3-year and H) 5-year OS in the validation cohort. (TIF 3422 kb) [file 12885_2019_5703_MOESM1_ESM.tif]
