# Supplementary material for: Development and validation of nomograms predicting survival in Chinese patients with triple negative breast cancer
Source: BMC Cancer. 2019 Jun 6;19:541. doi: 10.1186/s12885-019-5703-4 (PMC6555047; doi:10.1186/s12885-019-5703-4)
Supplement: Supplementary file 2 — Figure S1. Point assignment and prognostic score for DFS and OS. (DOCX 15 kb) [file 12885_2019_5703_MOESM2_ESM.docx]

Supplementary table 1 Point assignment and prognostic score for DFS and OS

| Variable | DFS_score | Estimated 5-Year DFS (%) | Variable | OS_score | Estimated 5-Year OS (%) |
| --- | --- | --- | --- | --- | --- |
| Tumor size |  |  | Tumor size |  |  |
| ≤2cm | 0 |  | ≤2cm | 0 |  |
| 2-5cm | 4.9 |  | 2-5cm | 6.8 |  |
| ＞5cm | 9 |  | ＞5cm | 10 |  |
| Node status, |  |  | Node status, |  |  |
| N0 | 0 |  | N0 | 0 |  |
| N1 | 3.7 |  | N1 | 4.8 |  |
| N2+N3 | 10 |  | N2+N3 | 9.9 |  |
| Ki67 index |  |  | Ki67 index |  |  |
| <40% | 0 |  | <40% | 0 |  |
| ≥40% | 5.9 |  | ≥40% | 6.8 |  |
| sTIL group |  |  | sTIL group |  |  |
| 0 | 8.9 |  | 0 | 9.2 |  |
| 10-19 | 2.7 |  | 10-19 | 5.2 |  |
| 20-49 | 1.8 |  | 20-49 | 2.1 |  |
| ≥50 | 0 |  | ≥50 | 0 |  |
| Total OS score |  |  | Total OS score |  |  |
| 0-14 |  |  | 0-14 |  | 95.6% |
| 15-20 |  |  | 15-20 |  | 85.5% |
| >20 |  |  | >20 |  | 62.6% |
| Total DFS score |  |  | Total DFS score |  |  |
| 0-10 |  | 87.0% | 0-10 |  |  |
| 11-17 |  | 70.2% | 11-17 |  |  |
| >17 |  | 40.6% | >17 |  |  |

Abbreviations: DFS, disease-free survival; OS, overall survival; sTIL, stromal tumor-inﬁltrating lymphocyte
